# Supplementary material for: Observation of an Alice ring in a Bose–Einstein condensate
Source: Nat Commun. 2023 Aug 29;14:5100. doi: 10.1038/s41467-023-40710-2 (PMC10465595; doi:10.1038/s41467-023-40710-2)
Supplement: Supplementary file 1 — Supplementary Information [file 41467_2023_40710_MOESM1_ESM.pdf]

Supplementary Information for

Observation of an Alice Ring in a Bose–Einstein Condensate

Alina Blinova, Roberto Zamora-Zamora, Tuomas Ollikainen, Markus Kivioja, Mikko Möttönen, and David S. Hall

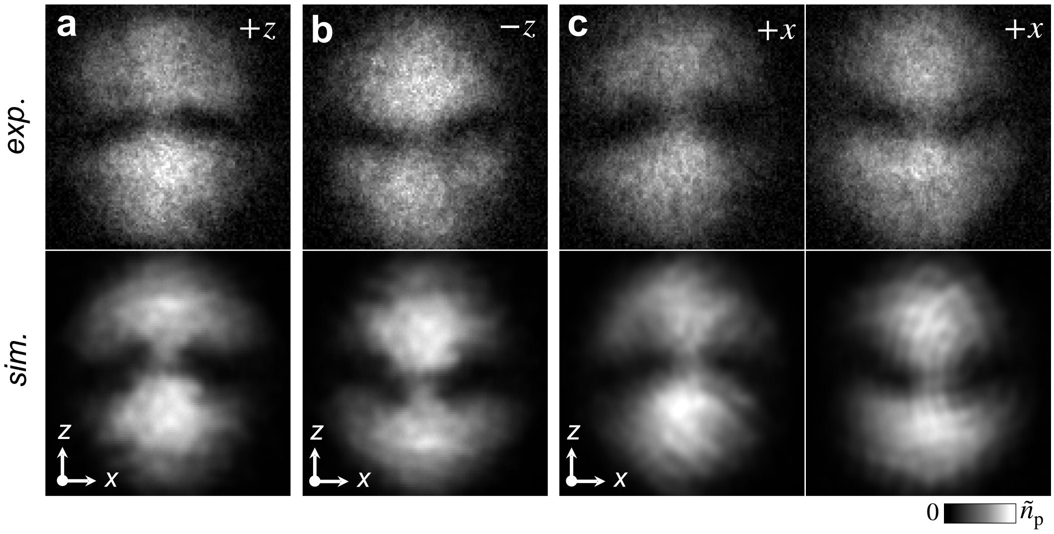

Supplementary Figure 1 | Experimental fingerprints of superfluid circulation about the Alice ring with the initial monopole created from different directions. a–c, Experimental (top) and simulated (bottom) column particle densities $\tilde{n}$ of the $m = 0$ spinor component viewed from the side at $T = 4$ ms after the field zero is brought into the condensate from the $+z$ direction (**a**), the $-z$ direction (**b**), and the $+x$ direction (**c**), with the left column in **c** corresponding to $T = 6$ ms. The densities are determined by the orientation of the initial bias field $\mathbf{B}_b$ before it is decreased to zero during the creation process. The simulations show that the difference in shape of the gap in the $m = 0$ component densities in **a** and **b** results from opposite windings of the scalar phase about the Alice ring. In these two cases the direction from which we create the initial monopole determines the sense of the phase winding. In the two cases shown in **c**, the field zero enters the condensate from the $+x$ direction, which can result in either sense of the superfluid circulation, as illustrated by the difference in the density profiles between the left and right columns. For each panel, the field of view is 219×219 μm^2 and the peak column density is $\tilde{n}_\text{p} = 6.89 \times 10^8$ cm^{-2}.

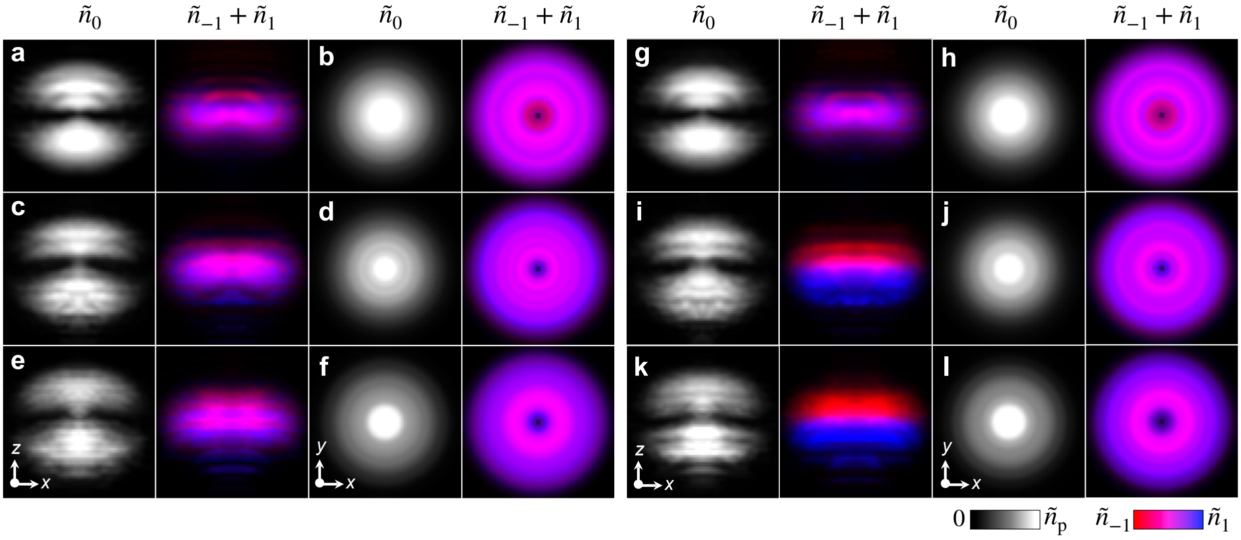

Supplementary Figure 2 | In-trap simulation results showing the effect of the magnetic field quench. **a**–**f**, Simulated column particle densities $\tilde{n}$ of a BEC evolving in the optical trap after the quadrupole contribution to the magnetic field is removed instantaneously. The $m = 0$ component and the combined $m = 1$ (blue) and $m = -1$ (red) components are shown for $T = 0$ ms (**a**,**b**), $T = 3$ ms (**c**,**d**), and $T = 5$ ms (**e**,**f**). The overlapping $m = \pm 1$ spinor components indicate that the interior region of the condensate remains almost completely in the polar phase. **g**–**l**, Same as **a**–**f** but with the experimentally achievable magnetic field quench. In this case the $m = \pm 1$ spinor components experience differential forces and separate along the z-axis, resulting in the partially polarised mixed-phase regions that are observed numerically and experimentally after expansion (Fig. 2c,e). For each panel the field of view is 12.4×12.4 μm^2 and the peak column density is $\tilde{n}_\mathrm{p} = 4.32 \times 10^{11}$ cm^{-2}.

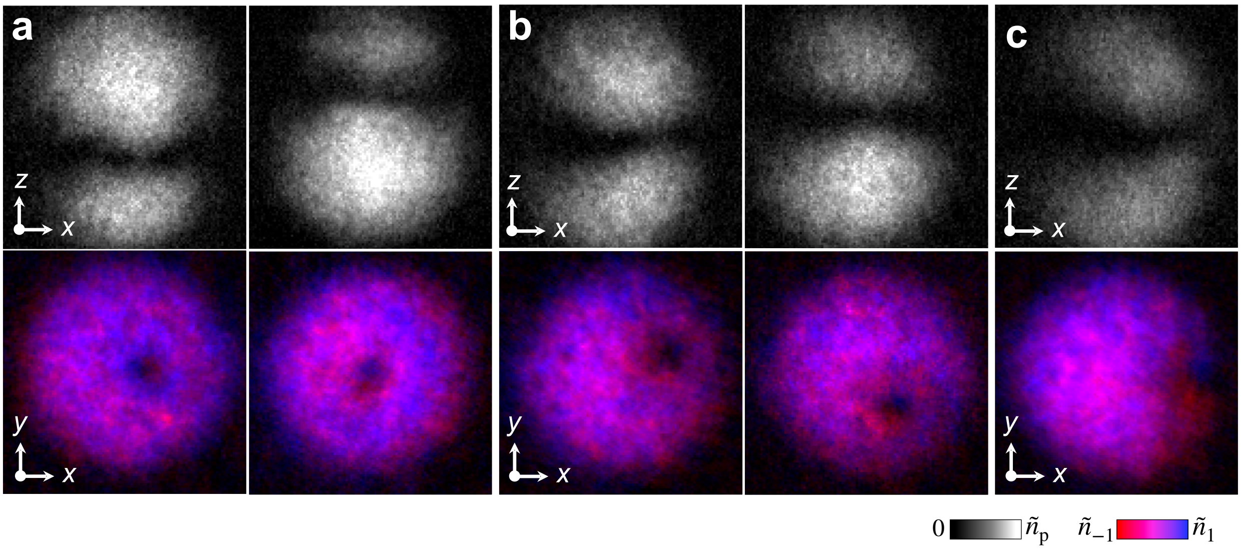

Supplementary Figure 3 | Monopoles created off-centre. **a**, Two examples of a monopole displaced in the vertical direction, with $m = 0$ component density in the top row (side view) and $m = \pm 1$ component densities in the bottom row (top view). The vertical shift of the monopole centre toward $-z$ (left column) and $+z$ (right column) is visible in the shift of the depleted region in the $m = 0$ component density. **b**, As **a** but for a monopole displaced horizontally in the $+x$ and $+y$ directions (left column) and in the $+x$ and $-y$ directions (right column). The horizontal shift is apparent in the displacement of the $m = \pm 1$ vortex centres. **c**, As **a** and **b** but for a monopole significantly displaced in the $+x$ direction. Even in the case of extreme displacement the component density distributions corresponding to the monopole are retained. For each panel, the field of view is $219 \times 219\ \mu\text{m}^2$ and the peak column density is $\tilde{n}_\text{p} = 6.89 \times 10^8\ \text{cm}^{-2}$.

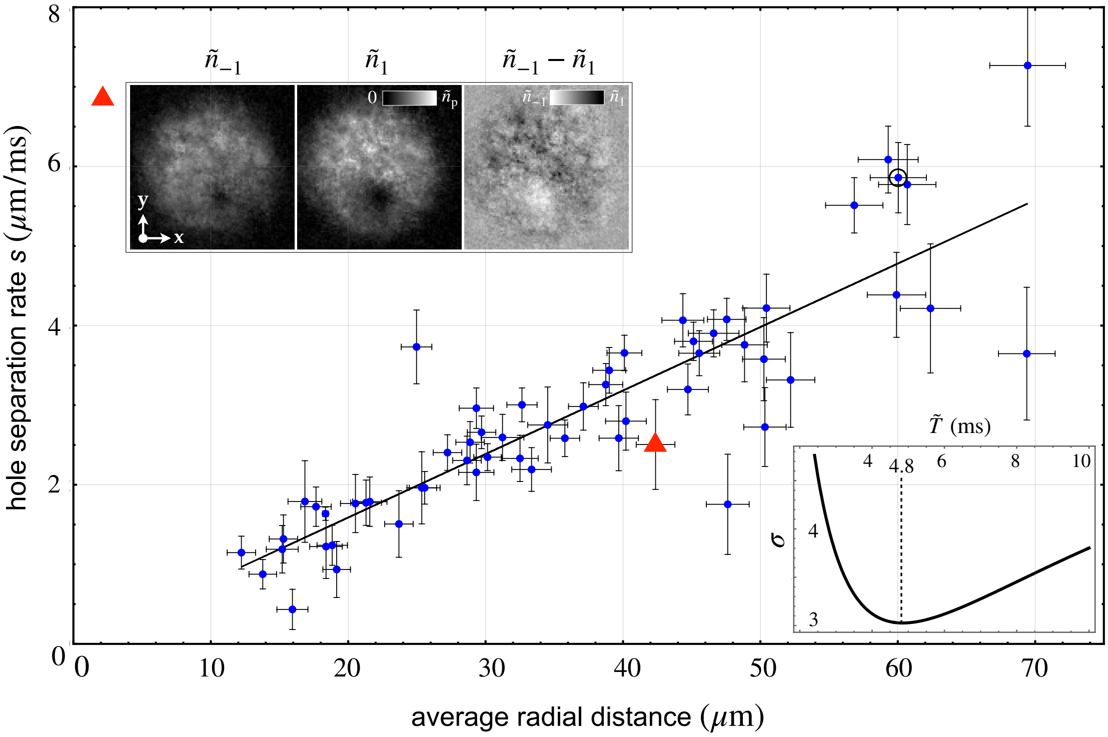

Supplementary Figure 4 | Growth of the Alice ring. Experimentally observed rate of separation s (blue dots) of the $m = \pm 1$ component density hole centres, from images taken at times $0 < T < 6$ ms, as a function of their average distance from the condensate centre. Error bars represent standard error of the fit to determine hole locations. The rate of separation is defined as the observed distance between the fitted hole centres divided by the total time $\tilde{T} + T$, where $\tilde{T}$ is a free parameter that takes into account the experimental observation that the hole separation at the edge of the condensate is nonzero for $T = 0$ ms, which suggests that the Alice ring is forming before the creation ramp is complete. An example of the separated hole centres at $T = 0$ is shown in the top left inset for the data point marked with a red triangle. The solid line is a linear fit to the data, giving the best estimate of the rate $\bar{s}$ for a given $\tilde{T}$. The value $\tilde{T} = 4.8$ ms minimises the relative variance $\sigma = \sum(\bar{s} - s)^2/\bar{s}^2$, as shown in the top left inset. Our simulations confirm the presence of a small Alice ring 4.8 ms before $T = 0$. The circled data point represents the case shown in Fig. 4e.

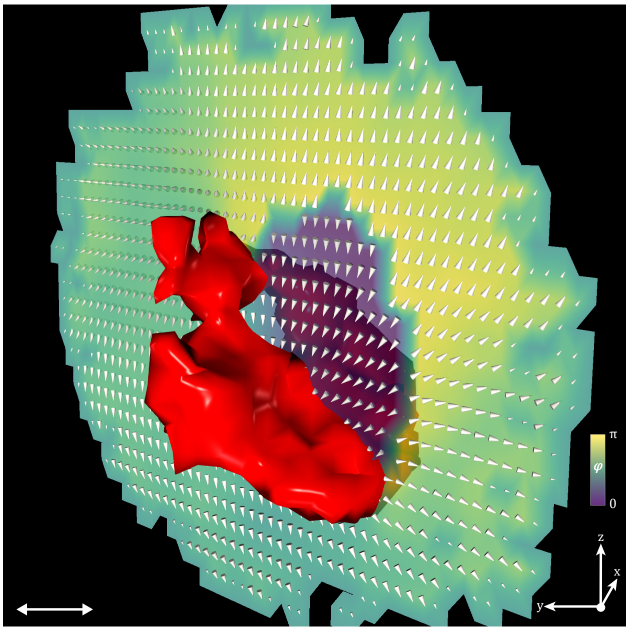

Supplementary Figure 5 | Long-lived Alice ring obtained by numerical simulations. The ferromagnetic core $|\langle \mathbf{F} \rangle| \geq 0.95$ (red surface) of the Alice ring remains visible after a simulated evolution time of $T = 84$ ms. The director field $\hat{\mathbf{d}}$ (white cones) and the scalar phase φ (background colour) are shown in the $x = y$ plane. The scale bar denotes 1 μm. Since the condensate is depicted in the trap, the field of view 8×8 μm^2 is substantially smaller than in the other figures of this work.
